# Supplementary material for: HIVprotI: an integrated web based platform for prediction and design of HIV proteins inhibitors
Source: J Cheminform. 2018 Mar 9;10:12. doi: 10.1186/s13321-018-0266-y (PMC5845081; doi:10.1186/s13321-018-0266-y)
Supplement: Supplementary file 2 — Additional file 2. Source code of HIVProtI web server. [file 13321_2018_266_MOESM2_ESM.zip › HIVprotI_Source-code/hivprotI/weka-3-6-6/doc/weka/gui/sql/event/package-frame.html]

weka.gui.sql.event


weka.gui.sql.event

Interfaces 
  
*ConnectionListener*
  
*HistoryChangedListener*
  
*QueryExecuteListener*
  
*ResultChangedListener*

Classes 
  
ConnectionEvent
  
HistoryChangedEvent
  
Messages
  
QueryExecuteEvent
  
ResultChangedEvent
